# Revival of Archaeal Methane Microbiology

**DOI:** 10.1128/mSystems.00181-17

**Published:** 2018-03-20

**Authors:** Cornelia U. Welte

**Affiliations:** a Department of Microbiology, Institute for Water and Wetland Research, Radboud University, Nijmegen, The Netherlands; b Soehngen Institute for Anaerobic Microbiology, Radboud University, Nijmegen, The Netherlands

**Keywords:** *Archaea*, methane, carbon cycle

## Abstract

The methane concentration in the Earth’s atmosphere is rising, and, as methane is a potent greenhouse gas, it contributes considerably to climate change. It is produced by methanogenic archaea that thrive in anoxic habitats and can be oxidized by methane-oxidizing bacteria or archaea. In this *Perspective*, recent innovations and discoveries in archaeal methane microbiology are discussed and a future outlook on how novel methane-metabolizing archaea might be cultivated is provided.

## Abstract (precis)

The methane concentration in the Earth’s atmosphere is rising, and, as methane is a potent greenhouse gas, it contributes considerably to climate change. It is produced by methanogenic archaea that thrive in anoxic habitats and can be oxidized by methane-oxidizing bacteria or archaea.

## PERSPECTIVE

In the past few years, the field of methane cycle microbiology has (re)gained interest due to many spectacular discoveries of novel archaea (and bacteria) involved in the production and conversion of the greenhouse gas methane. Forty years after the initial description of archaea (1), there is also a renewed interest in archaea in general. For decades, all archaea involved in the turnover of methane were affiliated with the Euryarchaeota. This group, called "methanogens," produces methane from a relatively restricted set of substrates such as H_2_ plus CO_2_, methylated compounds, and acetate. The members of the majority of methanogenic orders (Methanomicrobiales, Methanobacteriales, Methanococcales, Methanopyrales, Methanocellales) are able to perform hydrogenotrophic methanogenesis, where CO_2_ is reduced in a stepwise manner to methane with H_2_ as the reductant. Members of the order Methanosarcinales have a broad substrate spectrum and grow by reducing the methyl groups of methanol, methylated amines, or (rarely) methylsulfide to methane with concomitant oxidation of some of the methyl groups to CO_2_. For this, they partially reverse the methanogenesis pathway in order to obtain reducing equivalents to fuel their anaerobic respiratory chain. Only two methanogenic genera, *Methanosarcina* and *Methanosaeta*, are able to grow with acetate as the methanogenic substrate (2). They dismutate acetate to CO_2_ and methane, and even though they exhibit low growth rates and low phylogenetic diversity compared to other methanogens, it is estimated that they are responsible for about two-thirds of global biogenic methane production (3). Acetate can, however, also be converted to methane syntrophically by the action of acetate oxidizers that convert acetate to H_2_ and CO_2_ and subsequent conversion to methane by hydrogenotrophic methanogens. Irrespective of the pathways that methanogens use, they fulfill a paramount role in global nutrient cycling. Substrates for methanogenesis are compounds produced during the degradation of organic matter. As a consequence of their activity in turning those substrates into methane gas that is further converted to CO_2_, methanogens are an indispensable part of the global carbon cycle.

Despite the fact that strictly anaerobic methanogenic archaea cannot easily be cultivated in the laboratory, a large number of isolates have been described. However, recent advances in sequencing technologies revealed that the diversity of (potential) methanogens is far greater than previously thought, and many of those lineages have not yet been cultivated.

During the last 5 years, new methanogens were discovered with often quite surprising metabolic features. Novel methanogens were discovered within the Euryarchaeota, e.g., the Methanonatronarchaeia and Methanofastidiosa (4, 5). But even outside the Euryarchaeota, new potentially methanogenic microorganisms, for example, Verstraetearchaeota and Bathyarchaeota, have been discovered, mainly on the basis of metagenome-assembled genomes (6, 7). Among the euryarchaeotic groups, the Methanonatronarchaeia were found to thrive as methyl-reducing methanogens that are phylogenetically distant from the Methanosarcinales but instead closely related to the nonmethanogenic haloarchaea. Several isolates affiliated with the Methanonatronarchaeia are available in axenic culture. They are found in hypersaline environments and, just like the haloarchaea but unlike other methanogens, use the salt-in strategy for osmoprotection. Formate or hydrogen can be used as a reductant, as they are apparently incapable of oxidizing methyl groups to CO_2_ via partial reversal of the methanogenic pathway. For the Methanofastidiosa, no cultures were obtained but only genomic data exist. Metagenome-assembled genomes from the Methanofastidiosa revealed that they are probably methylsulfide-reducing methanogens connecting the carbon and the sulfur cycle. They seem to lack many biosynthesis pathways for amino acids as well as carbon and nitrogen fixation, characteristics which make them fastidious and therefore difficult to isolate in pure culture. One avenue in “culturing the unculturables” may be the construction of specific growth media based on the information obtained from metagenome-assembled genome sequences such as in the case of Methanofastidiosa. Enrichment or cultivation will enable exciting insights into these unique methanogens. There were also novel methanogens isolated from the human intestinal tract (8) that are closely related to the Thermoplasmata: the Methanomassiliicoccales. They thrive through the reduction of methylated compounds with hydrogen as the reductant, and, again, they are not affiliated with the Methanosarcinales. Besides interesting adaptations of their anaerobic respiratory chain (9), it has been found that they are widely distributed in the environment, including not only digestive tracts of many animals but also soils and sediments (10). Since members of this novel group are also associated with the gastrointestinal tracts of animals, including humans, research is dedicated to investigation of their potential role in health and disease and has even primed the idea of the use of archaea as probiotics (“archaebiotics”) (11).

A whole new group of archaea has been discovered that is closely related to methanogens but does not produce methane. Instead, the anaerobic methanotrophic (ANME) archaea use methane as the substrate via the pathway of reverse methanogenesis and oxidize it to carbon dioxide. The first ANME archaea to be discovered were those that dominate methane seeps at the sea floor, where they oxidize methane in consortia with sulfate-reducing bacteria (12). The exact mechanism of this interaction and many intriguing physiological and biochemical questions remain to be investigated. Consequently, in contrast to the many lineages of methanogens that are available in pure culture, the ANME archaea remain elusive and no axenic cultures are available yet. The most recent discovery is the ANME-2d group, whose members thrive not with sulfate but with nitrate (13) and oxidized metals as electron acceptors (14) in the absence of a bacterial syntrophic partner. However, cultivation and biochemical characterization of these ANME archaea have been started only recently (15) (unpublished results), and most ANME archaea remain elusive with respect to cultivation even though they are widely distributed in many different environments (16) and their genomes relatively well analyzed (17). However, to really understand the functioning and ecology of microorganisms, cultivation in the form of enrichment cultures and biochemical characterization are necessary. What could be new strategies for cultivating the unculturables from the methane cycle, beside the already mentioned use of genomic information to elucidate growth requirements? To answer this question, it is interesting to look at other approaches and innovations recently applied in the methane cycle, including the discovery of methoxydotrophic methanogenesis in the genus *Methermicoccus* (18, 19). These methanogens degrade methoxylated aromatic compounds (such as those found in coal) to methane by reducing the methoxy moieties to methane and excreting the respective aromatic compounds into the medium. Even though the methoxylated aromatic compounds are not fully mineralized by methoxydotrophic methanogens, it is astonishing that individual species utilize more than 30 different compounds for methanogenesis, which is the most extensive metabolic flexibility ever found in a methanogen. Previously, it was thought that such demethoxylation reactions would be catalyzed exclusively by syntrophic consortia of acetogens and methanogens. Yet, in the present study, it was clear that a single archaeon performed this whole reaction, even though it had been isolated using not methoxylated compounds but instead the rather conventional substrate methanol. Unification of the forms of metabolic labor of syntrophic microorganisms into a single microbial species was also found in comammox ("complete ammonia oxidizer") bacteria, which catalyze the complete oxidation of ammonium to nitrate, a process otherwise split into two steps by ammonia- and nitrite-oxidizing microorganisms (20, 21). According to the kinetic theory of optimal design of metabolic pathways, metabolic pathways are maximized for the rate of ATP production. The production of enzymes and the maintenance of intermediates are costly, so longer pathways may result in a decrease in individual enzyme and intermediate concentrations that leads to slower substrate turnover but may be accompanied by more ATP-generating steps, leading to higher growth yields (22). As a result, shorter metabolic pathways with higher enzyme and intermediate concentrations lead to higher growth rates at the cost of growth yield, as shorter pathways probably have fewer ATP-generating steps. Therefore, it is clear that under conditions of high substrate concentrations, the microbial division of labor prevails, whereas under diffusive, substrate-limited conditions, such as those probably encountered by comammox bacteria and methoxydotrophic methanogens, this picture could change. Their longer metabolic pathways make them competitive under conditions of such low substrate concentrations, where the growth yield determines the outcome of the competition (22). Past cultivation efforts were largely focused on batch or plate cultivation with high substrate concentrations, but it is now becoming more evident that a large amount of the total proportion of cultivated microorganisms may belong to the group with shorter metabolic pathways, neglecting a potentially large group of bacteria and archaea. Many environments exhibit extremely low substrate seepage through either diffusion or food chain dependencies; thus, the majority of microorganisms might rather be adapted to growth with a limited substrate supply. Furthermore, many microorganisms grow in biofilms on a variety of surfaces rather than in suspended culture. The latter could also be regarded as a specific adaptation to laboratory cultivation conditions. Hence, it becomes more and more evident that there is a need for a change of or diversification in cultivation strategies. In terms of methane cycle microorganisms, it might be possible to exploit this strategy using low substrate levels to enrich for methane-producing microorganisms with longer metabolic pathways, potentially converting peptides (e.g., Bathyarchaeota) or carbohydrates (e.g., Verstraetearchaeota) directly to methane. Continuous bioreactor cultivation combined with inocula from diffusive systems might be the key to cultivation of such elusive microbes of the methane cycle in the future to capture the full diversity of this interesting and ecologically important group of microorganisms.
